# A Dictionary

**Published:** 1876-12

**Authors:** 


### *A DICTIONARY.*

There are hundreds of thousands of homes in America without a dictionary worthy of the name, if indeed they have a dictionary of any kind. How parents can expect children to grow up with any accurate knowledge of the structure and use of words, without providing a good dictionary and encouraging them to appeal to it as often as a doubt arises as to the orthography or meaning of a word, we are at a loss to determine. The best scholars, writers and orators provide themselves with lexicons of various languages, and especially of the one which they use most constantly—their own. Few persons, and certainly no young man or woman, can trust themselves to employ or even to spell words not in common use, with entire accuracy, save by reference to that final court of arbitration, "Webster." Perhaps the best evidence of culture, the surest indication of broad acquisitions of knowledge, are found in good pronounciation, accurate spelling, and the ready and appropriate use of a large vocabulary. Now all this may be gained, in time, by reading the best books, by conversation with cultivated persons, by attending lectures and the like. Still, nothing takes the place of the dictionary, as every scholar knows. We have a Webster's Unabridged, which we would not sell for its weight in gold if we could not replace it.

